# Effect of *γ* radiation processing on fungal growth and quality characteristcs of millet grains

**DOI:** 10.1002/fsn3.295

**Published:** 2015-10-17

**Authors:** Nagat S. Mahmoud, Sahar H. Awad, Rayan M. A. Madani, Fahmi A. Osman, Khalid Elmamoun, Amro B. Hassan

**Affiliations:** ^1^ Environment and Natural Resource and Desertification Research Institute (ENDRI)National Center for ResearchPO Box 6096KhartoumSudan; ^2^ Sudanese Atomic Energy Commission (SAEC)KhartoumSudan

**Keywords:** Antinutritional factors, fungal growth, germination, millet, protein solubility, radiation

## Abstract

The aim of this study was to evaluate the effect of gamma radiation processing of millet grains on fungal incidence, germination, free fatty acids content, protein solubility, digestible protein, and antinutritional factors (tannin and phytic acid). The grains were exposed to gamma radiation at doses 0.25, 0.5, 0.75, 1.0, and 2.0 kGy. Obtained results revealed that radiation of millet grains at a dose level higher than 0.5 kGy caused significant (*P* < 0.05) reduction on the percentage of fungal incidence and the free fatty acid of the seeds, while, no significant change in the germination capacity was observed of the grains after radiation. Additionally, the radiation process caused significant (*P* < 0.05) reduction on both tannins and phytic acid content and gradual increment on in vitro protein digestibility of the grains. On the other hand, the treatments significantly (*P* < 0.05) increased the protein solubility of the grains. Obtained results indicate that gamma irradiation might improve the quality characteristics of millet grains, and can be used as a postharvest method for disinfestations and decontamination of millet grains.

## Introduction

Pearl millet (*Pennisetum gluucum* L.) is considered to be the staple food for most people in Asia and Africa. It is considered as a good source of needed elements (Abdalla et al. 1998). However, it contains high amounts of antinutrients such as tannin and phytic acids which reduce its nutritional value (Abdel Rahaman et al. 2007). During postharvest storage, millet is susceptible to attack by a variety of insects and microorganisms. This infestation cause physical losses and reduces the nutritional value of grains which leads to the loss of the economic value of stored grain. Moreover, infestation with the insects result in contamination with dead insect's bodies and their products, as well as fungal growth that favor the spread of *Aspergillus flavus*, a mold which produces aflatoxin (Rees 2004).

Generally, chemical fumigants are used to disinfest grains (Arthur 1996), however, continuous application of these pesticides have a negative impact either on the environment or human health (Cherry et al. 2005). Therefore, the industry has been forced to explore nonchemical alternatives. One possible alternative is the application of gamma irradiation. Radiation processing is considered to be a safe alternative to chemical methods, enhance quality and nutritional characteristics of stored products as well as maintain its shelf‐life. It has shown great promise in accomplishing disinfestations and decontamination of food and agricultural products (Loaharanu 1994; Fombang et al. 2005). Besides disinfection criteria, gamma radiations enhance the nutritional value of grains and improves the functional properties of its flours (Rahma and Mostafa 1988; Dario and Salgado 1994; Dogbevi et al. 2000). Furthermore, it has been reported that gamma radiation causes a significant reduction in antinutrients and enhances the nutritional quality of grains (Hassan et al. 2013; Osman et al. 2014). Although, application of gamma radiation has many advantages over other physical methods, however, the application of this technology in the industry is limited. Therefore, further research is needed to improve their efficiency to reach the reasonable usage stage and in controlling stored‐grain pests as well as to improve the nutritive value of stored products.

Thus, in the present study, gamma radiation was applied as a preserving method to investigate its efficiency in controlling fungal growth and enhancement of quality characteristics of millet grains.

## Material and Methods

### Sample preparation

Millet grains were cleaned manually and freed from broken seeds and impurities, and then stored in plastic bags at 4°C during the study.

### Radiation treatments

Radiation processing was done at Kaila irradiation processing unit, Sudanese Atomic Energy Corporation (SAEC). About 250 g of millet grains packed in polyethylene bags were irradiated, using a *γ*‐ ray ^60^Co radiator. The seeds were evenly exposed to radiation doses 0.25, 0.5, 0.75, 1.0, and 2.0 kGy with a dose rate of 33 Gy/min. Unirradiated seeds (0 kGy) served as control.

### Fungal culture and incidence

The fungal incidence and the colony formation unit per gram (cfu/g) of treated and untreated samples was determined after platted on double strength Sabaroud Dextrose Agar and incubated at 25°C for 5 days according to standard methods (AOAC 1995).

### Determination of germination

The germination of grains was determined according to the international Seed Testing Association (ISTA 2006). Twenty five seeds were platted on filter paper in a Petri dish and saturated with distilled water. The plates were incubated at 25 ± 2°C for 7 days.

### Determination of free fatty acid content

Free fatty acid of millet was determined according to Aibara et al. (1986) cited by Zhao et al. (2007) with slight modification. About 25 mL of ethanol was added to 5 g of millet flour. After shaking, the mixture was filtrated and additional 25 mL ethanol was added. The filtrate was titrated with 0.1 N KOH, using phenolphthalein (3%) as indicator. Flour acidity was calculated as mg KOH required neutralizing free fatty acid from one gram grain on dry matter basis.

### Determination of tannins and Phytic acid content

Tannins content of grains was estimated according to Price et al. (1978). Phytic acid content was determined by the method described by Wheeler and Ferrel (1971).

### Crude and digestible protein determination

The crude protein was determined following the Kjeldahl method described by AOAC (1995). The digestible protein was determined by the procedure of Maliwal (1983) cited from Monjula and John (1991).

$$\text{Protein digestibility}\;\%=\frac{\text{digestible protein}}{\text{total protein}}\times 100$$

### Protein solubility

Soluble protein solubility was determined in millet grains after extracted by water, using the method described by Hagenmaier (1972).

### Statistical analysis

All data were the average of triplicates. Data were analyzed using one‐way analysis of variance (ANOVA). Significant differences were calculated (*P* < 0.05), using least significant difference (LSD).

## Results and Discussion

### Effect of radiation process on the fungi incidence and germination rate

Table 1 presents the percentage of fungal incidence and colony formation (cfu/g) in raw and treated millet grains. Before radiation, the fungal incidence was found to be 100% in raw grains. Radiation of seeds up to 0.50 kGy caused no significant reduction in fungal incidence, however, radiation of grains at higher doses 0.75, 1.0 and 2.0 kGy sharply decreased the fungal incidence to 21.3, 18.7, and 5.3%, respectively. Similarly, the effectiveness of gamma radiation in reducing the formation of fungi was observed. Prior to radiation, the colony formation was found to be 5.3 × 10^4^ cfu/g, where as it was decreased to 2.1 × 10^4^, 2.1 × 10^4,^ 4 × 10^2,^ 3 × 10^2^ and 3 × 10 cfu/g after radiation treatment at doses of 0.25, 0.50, 0.75, 1.0, and 2.0 kGy, respectively (Table 1). Similar observation on walnut kernel was reported by Al‐Bachir (2004), who found that application of gamma radiation at doses of 0.5, 1.5, and 2 kGy reduced the fungal load on walnut kernels. Furthermore, it was reported that doses of 1.5 and 3.5 kGy reduced the number of fungi in many raw fruits and vegetables (Aziz and Moussa 2004). Hilmy et al. (1995) concluded that radiation process peanuts with doses up to 1 kGy inhibit the incidence of mycelium and toxins secretion. Reduction in the fungal incidence rate in millet grains after radiation might be due to high sensitivity of the fungus and mold to gamma radiation, since the radiation process causes direct and indirect damage to the DNA (Refai et al. 1996; McNamara et al. 2003).

**Table 1:** Effect of gamma irradiation on fungal growth (%) and colony formation (cfu/g) in pearl millet. Error bars indicate the standard deviation (*n* = 3). Values not sharing a common superscript are significantly (*P* < 0.05) different

Gamma dose (kGy)	Fungal incidence (%)	Colony formation (cfu/g)
0.0	100 ± (0.000)^a^	5.3 × 10^4^
0.25	100 ± (0.000)^a^	2.1 × 10^4^
0.50	100 ± (0.000)^a^	2.1 × 10^4^
0.75	21.3 ± (9.238)^b^	4.0 × 10^2^
1.0	18.7 ± (2.309)^b^	3.0 × 10^2^
2.0	5.3 ± (2.309)^c^	3.0 × 10^1^

**Figure 1:**
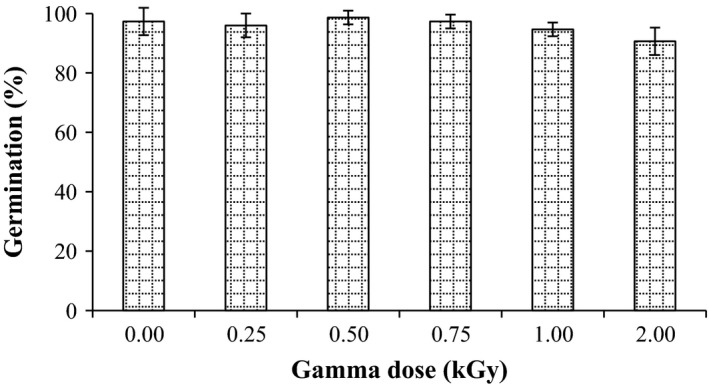
Effect of gamma irradiation on germination rate in pearl millet. Error bars indicate the standard deviation (*n* = 3).

Germination test is comparatively assessing the quality losses of grain after treatments. It is directly associated with various characteristics of grain quality (Beckett and Morton 2003). As shown in Figure 1, no significant change in seed germination rate after treatments was observed. Maximum decrease in germination rate of millet (90.7%) was observed with 2 kGy treatment. Obtained results were in accordance with El‐Naggar and Mikhaiel (2011), who found that the germination capacity of wheat grains was not changed after radiation at dose up to 1 kGy. Moreover, the results of Melki and Marouani (2009) showed that there was no significant change in germination capacity in wheat after radiation.

### Effect of radiation process on free fatty acids (FFA) content

**Figure 2:**
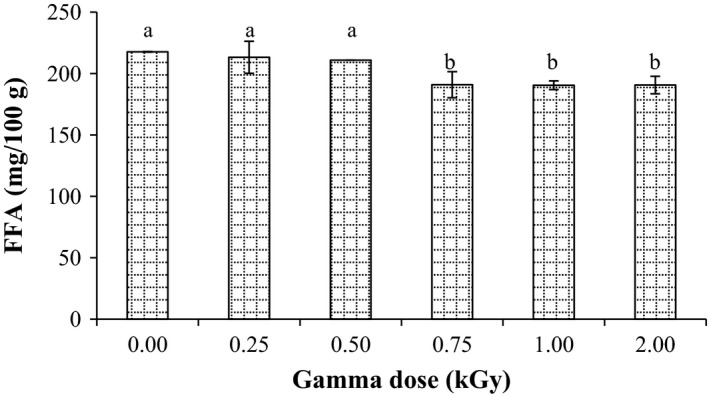
Effect of gamma irradiation on free fatty acids (FFA) in pearl millet. Error bars indicate the standard deviation (*n* = 3). Values not sharing a common superscript are significantly (*P* < 0.05) different.

Figure 2 presents the free fatty acids (FFA) content in mg/g in millet grains for the control and radiated samples. The FFA content of millet flour was found to be 217.7 mg/100 g prior to the radiation treatment. After radiation at dose levels of 0.75, 1.0, and 2.0 kGy, it is clearly observed that the FFA content of millet grains significantly (*P* < 0.05) reduced to 190.9, 190.6, and 190.5 mg/100 g, respectively. Significant reduction on FFA content might be due to lipase activity reduction in treated grains, which result in dropping the FFA formation. Pankaj et al. (2013) demonstrated that the radiation treatment significantly reduced the lipase activity in wheat germ. Therefore, the results indicated that gamma radiation is an effective method for stabilization and to extend the shelf life of grains, since free fatty acids content is an index of the rancidity and contributes to the development of off‐flavor and off‐ odors in oil during storage.

### Effect of radiation process on tannin and phytic acid content

**Figure 3:**
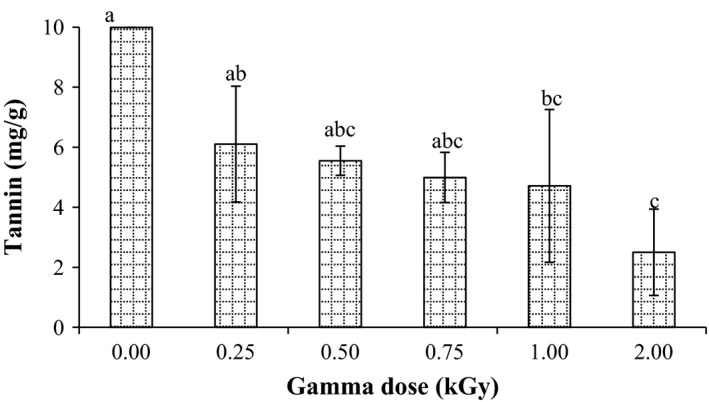
Effect of gamma irradiation on tannin content in pearl millet. Error bars indicate the standard deviation (*n* = 3). Values not sharing a common superscript are significantly (*P* < 0.05) different.

As illustrated in Figure 3, the tannin content of millet grains was found to be 9.99 mg/g prior to radiation process. Tannin content of the examined grains presented a dose‐dependent decrease. It was clearly observed that radiation significantly (*P* < 0.05) reduced tannin content of millet grains. The reduction in tannin content was found to be 38.9, 44.4, 50, and 52.8, and 74.9% when millet grains were irradiated at dose 0.25, 0.50, 0.75, 1.0, and 2.0 kGy, respectively, compared to control one. These findings are in agreement with those stated by several researchers. Hassan et al. (2009) concluded that radiation process significantly reduced tannins content of sorghum and maize grains. Similar observation was reported by Pinn (1992) who stated that radiation of white beans at dose levels 2, 4, 6, 8, 10, 15, and 20 kGy followed by cooking significantly reduced tannins content. Moreover, El‐Niely (2007) found that the tannin content of legume seeds decreased after radiation treatments. Decrease in tannin content might be result of chemical degradation by the action of free radicals formed by the radiation.

**Figure 4:**
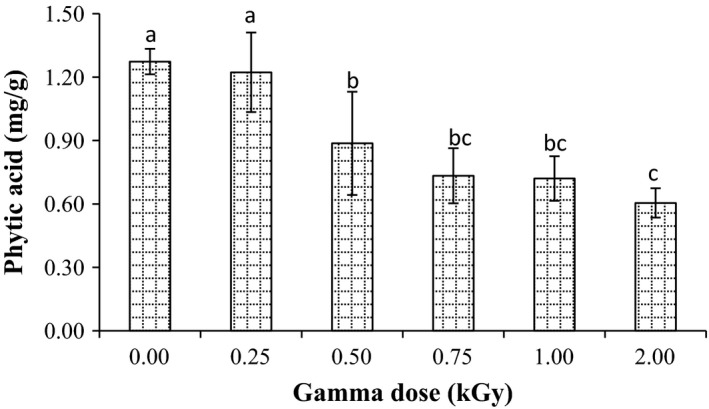
Effect of gamma irradiation on phytic acid content in pearl millet. Error bars indicate the standard deviation (*n* = 3). Values not sharing a common superscript are significantly (*P* < 0.05) different.

On the other hand, before radiation treatment, the phytic acid of millet grains was found to be 1.27 mg/g (Fig. 4). After radiation, phytic acid of millet grains significantly (*P* < 0.05) decreased. The level of reduction increased with an increase in radiation dose. Decreases in phytic acid were 3.9, 29.9, 42.5, 43.3, and 52.8%, at dose 0.25, 0.50, 0.75, 1.0, and 2.0 kGy, respectively. This reduction might be the result of the action of free radicals, since they are able to cleave to the phytate ring (De Boland et al. 1975). Obtained results were in agreement with El‐Niely (2007) who stated that radiation after processing significantly (*P* ≤ 0.05) decreased the level of phytic acid of legumes and cereal grains.

### Effect of radiation process on in vitro protein digestibility (IVPD) and protein solubility

**Figure 5:**
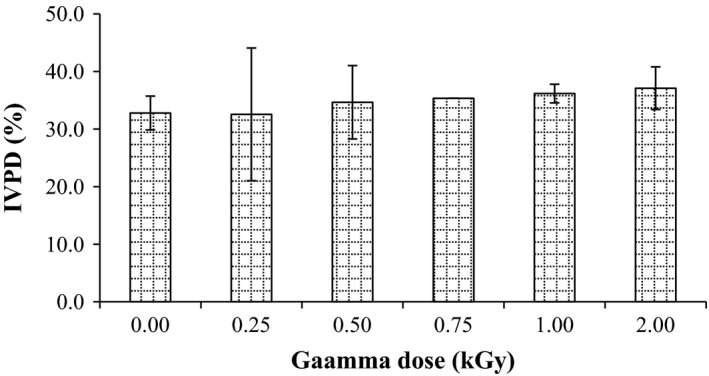
Effect of gamma irradiation on in vitro protein digestibility (%) in pearl millet. Error bars indicate the standard deviation (*n* = 3).

Figure 5 summarizes the in vitro protein digestibility (IVPD) of millet before and after radiation. The IVPD of untreated seeds was found to be 32.8%. Radiation process of the seeds caused a minor increment of the IVPD and was increased as the dose was increased. Increment in protein digestibility might be due to the reduction in the antinutrients particularly tannin content of grains as reported by Hassan et al. (2009). Since disulphide and hydrogen bonds are involved in stabilizing protein structure, their breaking can result in loss of conformational or structural integrity that exposed additional peptide bonds, thus enhancing proteolysis. Irradiation can cause change in their protein structure that enhances denaturation of the protein and hence improve its digestibility (Koppelman et al. 2005).

**Figure 6:**
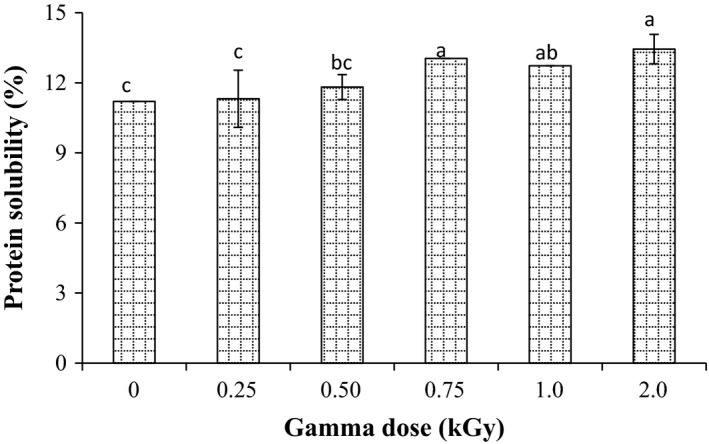
Effect of gamma irradiation on protein solubility in pearl millet. Error bars indicate the standard deviation (*n* = 3). Values not sharing a common superscript are significantly (*P* < 0.05) different.

Protein solubility is doubtless the most important function among the functional properties of proteins. Data in Figure 6 showed that the protein solubility of raw millet was found to be 11.20%. The protein solubility of millet was increased significantly (*P* < 0.05) to 11.32, 11.82, 13.04, 12.74, and 13.44% after radiation at 0.25, 0.50, 0.75, 1.0, and 2.0 kGy, respectively. Increase in protein solubility after radiation is likely due to the high proteolytic activity during radiation, which may lead to hydrolysis of the stored proteins.

## Conclusion

The obtained results revealed that gamma irradiation processing of millet grains up to 2 kGy significantly reduced the fungal incidence and free fatty acids content of the grains. On the other hand, it caused a decrease in the amount of antinutrients namely, tannin and phytic acid and gradually increase the in vitro protein digestibility and protein solubility of the grains. According to these results, therefore, gamma radiation can be applied as a safe postharvest method in order to extend the shelf life of millet grains.

## Conflict of Interest

None declared.
